# Clinical course of a Japanese patient with developmental delay linked to a small 6q16.1 deletion

**DOI:** 10.1038/s41439-022-00194-w

**Published:** 2022-05-17

**Authors:** Tetsuya Okazaki, Tatsuya Kawaguchi, Yusuke Saiki, Chisako Aoki, Noriko Kasagi, Kaori Adachi, Ken Saida, Naomichi Matsumoto, Eiji Nanba, Yoshihiro Maegaki

**Affiliations:** 1 Division of Clinical Genetics, Tottori University Hospital, Yonago, Japan; 2 Division of Child Neurology, Department of Brain and Neurosciences, Faculty of Medicine, Tottori University, Yonago, Japan; 3 Department of Fundamental Nursing, School of Health Science, Tottori University Faculty of Medicine, Yonago, Japan; 4 Research Initiative Center, Organization for Research Initiative and Promotion, Tottori University, Yonago, Japan; 5 Department of Human Genetics, Yokohama City University Graduate School of Medicine, Yokohama, Japan; 6 Research Strategy Division, Organization for Research Initiative and Promotion, Tottori University, Yonago, Japan

**Keywords:** Autism spectrum disorders, Neurodevelopmental disorders

## Abstract

There is only one report of patients with developmental delay due to a 6q16.1 deletion that does not contain the *SIM1* gene. A 3-year-old female showed strabismus, cleft soft palate, hypotonia at birth, and global developmental delay. Exome sequencing detected a *de novo* 6q16.1 deletion (chr6: 99282717–100062596) (hg19). The following genes were included in this region: *POU3F2, FBXL4, FAXC, COQ3, PNISR, USP45, TSTD3, CCNC*, and *PRDM13*.

Various rare copy number variations (CNVs) are associated with developmental delay and intellectual disability. Moreover, the progress of genetic analysis methods, including chromosomal microarray or next-generation sequencing, contributes to understanding these phenotypes. For example, patients with the 6q16.1-q21 deletion have been known to show a Prader Willi syndrome-like phenotype^1,2^. In these patients, the *SIM1* gene at 6q16.3 was suspected to be related to the obese phenotype. However, Kasher et al. described 10 patients from six families with obesity and variable developmental delay, with small deletions at 6q16.1 that did not contain the *SIM1* gene^3^. Due to the rarity of these cases, there is limited understanding of the clinical features of patients with the 6q16.1 small deletion that does not include *SIM1*. Therefore, we report the first Japanese patient with developmental delay due to this 6q16.1 small deletion.

A 3-year-old female was born at 38 weeks to nonconsanguineous parents. The family history included no reports of intellectual disability or developmental delay. Her birth weight was 2914 g (0.6 SD), her body length was 47.5 cm (−0.3 SD), her head circumference was 35.5 cm (1.5 SD), and there was no birth asphyxia. She showed strabismus, cleft soft palate, and hypotonia in the neonatal period. At 5 months of age, eye contact was difficult. At 6 months of age, she could control her head but rarely smiled. Additionally, at 7 months of age, she could turn over, and at 11 months of age, she could stand with support. At 1 year and 0 months old, G-banding and head MRI showed normal results. Surgical treatment for cleft palate was performed at 1 year and 3 months old. At 1 year and 5 months, she could walk with support but could not imitate or understand language. She was admitted to our hospital at 1 year and 9 months old to examine her developmental delay. Her body weight was 10.0 kg (−0.4 SD), her body height was 79.7 cm (−0.8 SD), and her head circumstance was 49.1 cm (1.5 SD). She revealed hypotonus, an attenuated deep tendon reflex in both lower limbs, and autistic features. However, muscle weakness, fasciculations, involuntary movements, and pyramidal signs were not noted. Echocardiography, auditory brainstem response, and peripheral nerve conduction studies revealed normal results. Her developmental quotient at this age assessed by the Enjoji Infantile Development Test was 40, and this score indicated moderate developmental delay (36–50). She could walk at the age of 2 years and 4 months and could speak meaningful words at 2 years and 9 months. At the age of 5 years and 1 month, she could not speak two-word sentences, her body weight was 15.7 kg (−0.8 SD), and her body height was 101.7 cm (−1.1 SD).

Furthermore, whole-exome sequencing was performed to determine the causative gene in the parents–patient trio after obtaining written and informed consent from the parents^4^. The examination protocols were approved by the Central Ethics Committee of Tohoku University School of Medicine Hospital (2018–2-216). Exome sequencing data were analyzed using the eXome Hidden Markov Model (XHMM) and modified Nord’s method^5^. We detected the 6q16.1 deletion (chr6: 99282717–100062596) (hg19) (Fig. 1). Both parents did not show this 780 kb deletion using the above methods. These CNVs have previously been reported in patients with developmental delay^3^. The following genes were included in this region: *POU3F2, FBXL4, FAXC, COQ3, PNISR, USP45, TSTD3, CCNC*, and *PRDM13*.

**Fig. 1:**
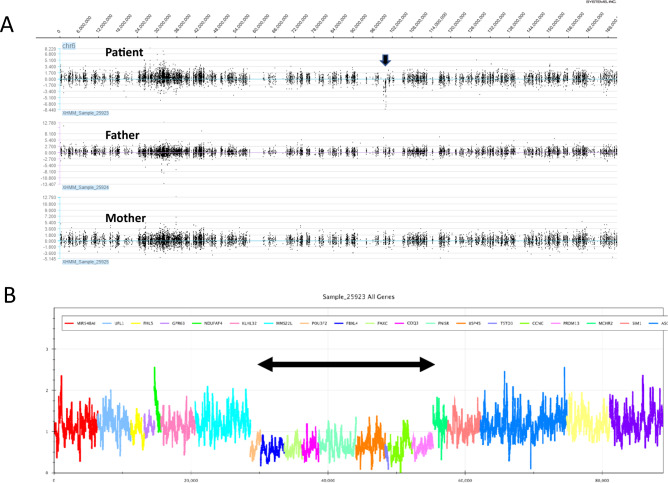
Graphic representation of copy number variation analysis. **A** Graphic representation of copy number variation analysis using an eXome Hidden Markov Model (XHMM). This image shows the deletion in 6q16.1 of this patient (arrow). However, neither parent showed this deletion. **B** Graphic representation of copy number variation analysis using a modified Nord’s method. This image shows the deletion in 6q16.1 (arrow).

Among the genes included in the deletion region of this patient, *FBXL4* and *USP45* were registered as causative genes of Mendelian disorders in Online Mendelian Inheritance in Man (OMIM)^6^. However, both genes are recessively inherited, disease-causing genes in which heterozygous carriers are phenotypically unaffected. The six families described in the report by Kasher et al. had the deletion of the *POU3F2* and *FBXL4* genes, although the deletion size was different in each family^3^. The etiology by which the deletion of this part of 6q16.1 causes this phenotype has not been clarified; however, it has been speculated that haploinsufficiency of the *POU3F2* gene is the cause of this phenotype. Additionally, in previously reported cases^3^, the onset of symptoms varied from early childhood to adulthood, but overeating and obesity were frequently observed. Nasu et al. described the possible involvement of the *POU3F2* gene in developmental delay and increased appetite in experiments from a mouse study^7^. Thus, we need to pay attention to her symptoms of obesity. Developmental delay and muscle hypotonia were described in previous reports, but strabismus and cleft palate were not described^3^. To the best of our knowledge, the association of each gene could not explain these phenotypes. Therefore, this genetic testing result may not explain these symptoms. We are the first to report that head MRI, peripheral nerve conduction studies, and auditory brainstem response are normal in patients with developmental delay due to a 6q16.1 deletion that does not contain the *SIM1* gene. Therefore, it is desirable to accumulate more case information in the future to improve the medical management of this disease.

## Data Availability

The relevant data from this Data Report are hosted at the Human Genome Variation Database at 10.6084/m9.figshare.hgv.3168. 10.6084/m9.figshare.hgv.3171. 10.6084/m9.figshare.hgv.3174. 10.6084/m9.figshare.hgv.3177. 10.6084/m9.figshare.hgv.3180. 10.6084/m9.figshare.hgv.3183. 10.6084/m9.figshare.hgv.3186. 10.6084/m9.figshare.hgv.3189. 10.6084/m9.figshare.hgv.3192.
